# Editorial: Using technology for healthy lifestyle and weight management

**DOI:** 10.3389/fendo.2022.973006

**Published:** 2022-08-04

**Authors:** Negar Naderpoor, Ayesha Barmare, Helen Skouteris, Brian Jack, Helena Teede

**Affiliations:** ^1^ Monash Centre for Health Research and Implementation, School of Public Health and Preventive Medicine, Monash University, Clayton, VIC, Australia; ^2^ School of Clinical Sciences, Monash University, Clayton, VIC, Australia; ^3^ Health and Social Care Unit, School of Public Health and Preventive Medicine, Monash University, Clayton, VIC, Australia; ^4^ Boston University School of Medicine, Boston, MA, United States

**Keywords:** digital health, weight management, lifestyle modification, technology, mobile applications

Technology is a double-edged sword when it comes to lifestyle and healthcare. Whilst technology can lead to sedentary behaviors and weight gain, it also has benefits such as greater accessibility and equity for lifestyle modification and health promotion. Traditional lifestyle and weight management interventions are often intense and time consuming hence proving difficult to integrate into routine clinical care. The recent Covid-19 pandemic has further revealed the advantage of technology in delivering health interventions especially when face to face interactions had to be limited.

This collection of articles presents a variety of strategies and implications of technology use for healthy lifestyle and weight management including adaptations to target specific populations. It also informs future research by identifying the gaps in the current literature.

A meta-review of 72 systematic reviews of Randomised Clinical Trials (RCTs) by Castro et al. demonstrated that smartphone apps and websites were the most frequently used methods of digital health interventions for improving lifestyle. Among the 6 domains of lifestyle, digital health interventions most commonly addressed diet and physical activity. Their review highlights that very few studies to date have investigated the role of digital interventions in other lifestyle aspects including substance use, stress management, sleep, and social relationships.

Digital health is a rapidly developing field with over 350,000 applications available globally (1). This vast number of options can leave clinicians and patients feeling inundated and overwhelmed. The review by Ghelani et al. serves to update and notify clinicians about the position of technology in the field of lifestyle and weight management. They summarise the findings from RCTs on the effectiveness of mobile apps in weight management and highlight the factors influencing their use. Several authors in this collection, including Ghelani et al. found loss of motivation over time and high attrition rates to be one of the main barriers in using mobile apps limiting the efficiency of technology for lifestyle modification and weight management and acting as a source of bias in research outcomes. While many studies have demonstrated the acceptability and effectiveness of apps in weight management, limitations such as small sample sizes, heterogeneity in apps and users as well as measured outcomes, and short follow up prevent generalisation of the findings.

New technology-based tools for data collection and analysis including recalls through websites, image-based or wearable devices and handheld personal digital assistants (PDA) allow faster and often cheaper data collection and analysis. Burrows et al., conducted a systematic review and examined the validity of these methods and other self-reported methods of dietary intake assessment with doubly labelled water (DLW). Their review demonstrated significant under-reporting of energy intake when a self-reporting method was used compared to DLW and the results were similar for technology-based methods. The handheld PDA and the Remote Food Photography Method for food records were shown to have the lowest rate of misreporting which may indicate their ability for more accurate diet recording.

Duan et al. presented the potential of social media in delivering lifestyle interventions to improve accessibility and convenience. Their study protocol uses WeChat, a popular social media in China, to deliver a home-based cardiac rehabilitation program to promote healthy lifestyle for those in remote areas.

Three of the articles in this collection investigate the role of technology in delivering lifestyle and weight management to women at different life phases. Women’s lifestyle during preconception, pregnancy, and postpartum periods have a substantial impact on maternal and neonatal outcomes.

Gardiner et al. introduced “Gabby”, an online conversational agent, designed to deliver culturally appropriate risk assessment and preconception nutritional and health education to African American women. In a secondary analysis of their RCT, they demonstrate the positive impact of this technology on behavioural change and improving nutrition and supplemental intake among 480 women over 6 and 12 months. The intervention was most beneficial to women with least nutrition knowledge at baseline and those who spent more time using Gabby. Ainscough et al. presented the results of PEARS (Pregnancy Exercise And nutrition Research Study) RCT including 565 pregnant women. They showcase the benefits of a smartphone app integration, developed by a multidisciplinary team, within routine antenatal care in Ireland to deliver the intervention content and improve maternal diet during pregnancy.

Among postpartum women, Lim et al., reported high acceptability of digital interventions for lifestyle modification. According to their systematic review of 9 qualitative studies, feedback and monitoring, goal setting, education, reminders, and peer support through an online forum were all perceived as valued digital health strategies by postpartum women. Personalization of intervention, delivery through social media, mobile apps instead of website-based apps, tracking and ability to print or email the information were suggested as potential features to improve the digital interventions. Notably, they found no studies investigating health professionals’ perspectives on digital interventions for postpartum lifestyle management. This illustrates a significant gap in the current literature given the importance of clinician engagement in the success of postpartum interventions for weight management (2).

Yang et al., presented the findings from a retrospective study analyzing the outcomes of a digital behavior change intervention for weight management (Noom program) across six English speaking countries including USA, Canada, UK, Ireland, Australia, and New Zealand. This intervention has been shown in observational and RCTS to be effective in achieving significant weight loss among healthy and at-risk groups. They demonstrated that the same mobile intervention could produce comparable weight loss results in different countries with the same language and similar attitudes towards food and physical activity. Although weight outcomes were comparable, there were differences in how people engaged with the app highlighting the importance of cultural and linguistic tailoring when introducing technology-based interventions for lifestyle and weight management. Like other studies, participants with higher levels of engagement with the app achieved better outcomes.

Weight management apps have high acceptability from users, with over 25,000 apps currently available. However, only a few (0.05%) have been developed using identifiable health professionals’ input (3). While many apps are advertised as highly effective in weight management, only a few have been studied and proven to assist significant weight loss. Individuals interested in using technology for weight management deserve to have access to the apps that are effective and substantiated by reliable evidence. To date, there is no system to identify or endorse technology products independently to ensure and communicate that they are evidence based. Furthermore, no clinical practice guideline is available on the role of technology in lifestyle and weight management. Future research in this field needs to 1) generate robust evidence on usefulness of available apps in lifestyle and weight management (4), 2) identify strategies to endorse and identify those apps that are evidence based, 3) personalise and improve consumers’ motivation and engagement with apps, which are essential to effectiveness, 4) integrate functionality to enable engagement of health professionals for coaching and or data sharing in clinical settings. Finally, a co-design approach engaging stakeholders, clinicians and consumers is imperative for effective integration of the technology assisted lifestyle and weight management interventions into clinical care (5).

## Author contributions

NN conceptualized and wrote the first draft of the manuscript. All authors, NN, AB, HS, BJ and HT contributed to editing and revision of the manuscript as well as approval of the submitted version.

## Conflict of interest

The authors declare that the research was conducted in the absence of any commercial or financial relationships that could be construed as a potential conflict of interest.

## Publisher’s note

All claims expressed in this article are solely those of the authors and do not necessarily represent those of their affiliated organizations, or those of the publisher, the editors and the reviewers. Any product that may be evaluated in this article, or claim that may be made by its manufacturer, is not guaranteed or endorsed by the publisher.
